# Supplementary material for: Identifying human encounters that shape the transmission of Streptococcus pneumoniae and other acute respiratory infections
Source: Epidemics. 2018 Dec;25:72–9. doi: 10.1016/j.epidem.2018.05.008 (PMC6227246; doi:10.1016/j.epidem.2018.05.008)
Supplement: Supplementary file 1 [file mmc1.docx]

# Supporting Information

## Additional Figures

###### Figure S1: Difference in the mean number of *physical* contact between pneumococcal carriers and non-carriers (Panel A) and between individuals suffering and not suffering from respiratory symptoms (Panel B)

######
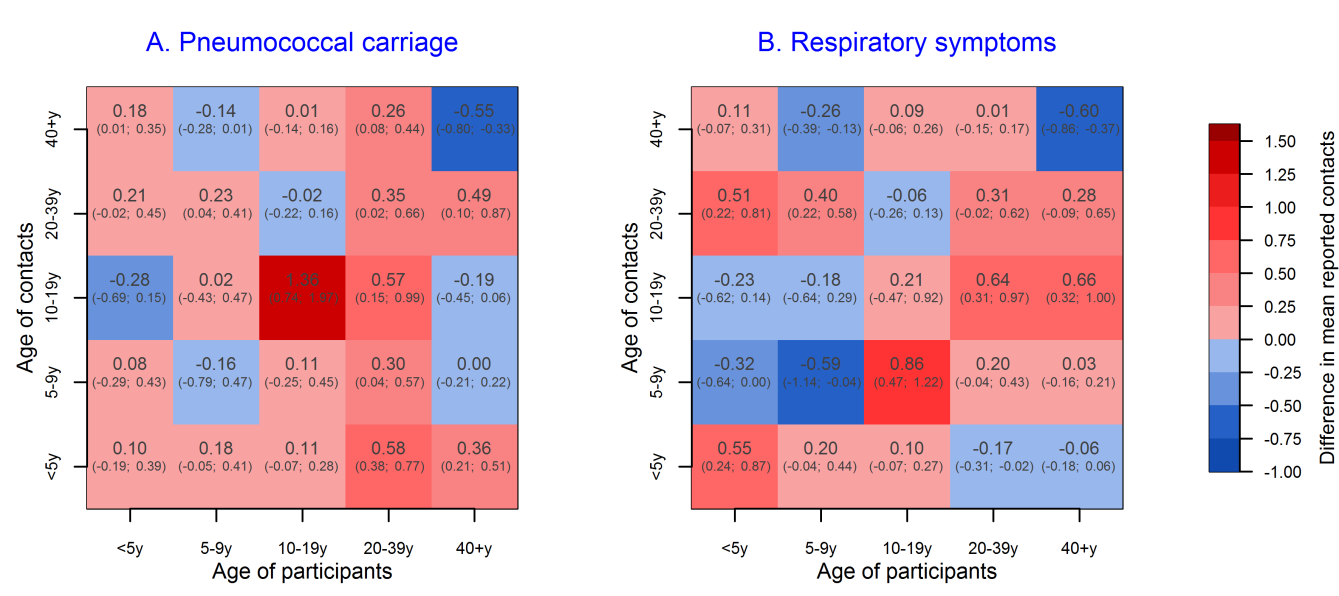


###### Legend: Matrices of the mean difference between the mean number of contacts among pneumococcal carriers and non-carriers (panel A), and individuals with ARS compared to non-ARS (panel B). The numbers represent the point estimate and the lower and upper bound of the 95% credible interval are shown inside the brackets.

## Analysis and model comparison

### Univariable analysis

In univariable analysis we explored the risk of pneumococcal carriage or ARS as a function of the following covariates: age, sex, and all types of ordinary contacts (all, physical (skin-to-skin), household, long (≥1h), non-physical, non-household, short (<1h)). We used a log-binomial regression model through a generalized linear model with a robust variance estimator, and inclusion probability weights by age group to capture the differences in inclusion by age at the sampling stage.

We considered contacts as linear predictors, but tested departure from linearity based on BIC values and likelihood ratio tests comparing models with contacts as linear variables and as categorical variables. On that basis there was no evidence of departure from linearity (BIC larger in all cases and P-value of likelihood ratio tests >P=0.10)

## Multivariable analysis

### Pneumococcal carriage

Age was first added to each model as an a priori confounding variable. Age-adjustment improved all models of pneumococcal risk as a function of all other covariates, leading to substantial changes in measures of effect (based on a lower BIC and a likelihood ratio test P-value <0.05).

We modelled the association between age-adjusted covariates and carriage through the following models:$\log(P_{AX})=\beta_{0}+\beta_{1}Age+\beta_{2}X$ , where A is Age and X was the covariate of interest (sex, or any type of ordinary contact (all, physical, etc.))

Only all contacts, physical contacts, household contacts and long (≥ 1h) contacts were associated with carriage after age adjustment. There was a strong correlation between physical contacts and contacts lasting more than one hour (Pearson’s correlation coefficient R=0.76), particularly in children <10 years of age (R>0.85), hence those covariates were not added together in a model due to collinearity. For household contacts correlation was moderate with physical contacts (R=0.61) or long contacts (R=0.64) and we therefore explored models including both physical contacts and household contacts, as well as contacts ≥1h and household contacts, in order to explore their potential confounding effect.

Table S1 provides the BIC values for the different models tested for the association with physical, household and long contacts. We found that model included a single type of contact as covariate performed better than those including two or more, hence only age-adjusted estimates were presented in the main paper.

###### Table S1: Comparing the BIC for models of pneumococcal carriage risk as a function of physical, household and long contact.

| **Model [covariates]** | **BIC** |
| --- | --- |
| $\log(P_{P})=\beta_{0}+\beta_{1}P$ [P=Physical contacts] | -3218 |
| $\log(P_{H})=\beta_{0}+\beta_{1}H$ [H=Household contacts] | -3199 |
| $\log(P_{L})=\beta_{0}+\beta_{1}L$ [L=Long (≥1h) contacts] | -3202 |
| $\log(P_{AP})=\beta_{0}+\beta_{1}A+\beta_{2}P$ [A=Age, Physical contacts] | -3312 |
| $\log(P_{AP})=\beta_{0}+\beta_{1}A+\beta_{2}H$ [Age, Household contacts] | -3310 |
| $\log(P_{AP})=\beta_{0}+\beta_{1}A+\beta_{2}L$ [Age, Long (≥1h) contacts] | -3312 |
| $\log(P_{APH})=\beta_{0}+\beta_{1}A+\beta_{2}P+\beta_{3}H$ [Age, Physical, Long] | -3306 |
| $\log(P_{ALH})=\beta_{0}+\beta_{1}A+\beta_{2}L+\beta_{3}H$ [Age, Long, Household] | -3306 |

In bold: best models

### ARS

The same approach as for pneumococcal carriage was taken. Again, age was considered as the main a priori confounding factor, given the differences in both carriage frequency and ARS prevalence by age. However, there was no improvement in model performance (no change or increase in BIC and non-significant likelihood ratio test) and little or no change in point estimates. Adding other variables to the model resulted in worse model performance.

Table S2 shows the different models explored for covariates associated with ARS at P<0.05 in univariable analysis (Physical contacts, household contacts, and contacts >1h).

###### Table S2: Models explored for ARS

| **Model [covariates]** | **BIC** |
| --- | --- |
| $\log(P_{P})=\beta_{0}+\beta_{1}P$ [P=Physical contacts] | -3288 |
| $\log(P_{H})=\beta_{0}+\beta_{1}H$ [H=Household contacts] | -3286 |
| $\log(P_{L})=\beta_{0}+\beta_{1}L$ [L=Long (≥1h) contacts] | -3286 |
| $\log(P_{AP})=\beta_{0}+\beta_{1}A+\beta_{2}P$ [A=Age, Physical contacts] | -3285 |
| $\log(P_{AP})=\beta_{0}+\beta_{1}A+\beta_{2}H$ [Age, Household contacts] | -3286 |
| $\log(P_{AP})=\beta_{0}+\beta_{1}A+\beta_{2}L$ [Age, Long (≥1h) contacts] | -3285 |
| $\log(P_{APH})=\beta_{0}+\beta_{1}A+\beta_{2}P+\beta_{3}H$ [Age, Physical, Long] | -3279 |
| $\log(P_{ALH})=\beta_{0}+\beta_{1}A+\beta_{2}L+\beta_{3}H$ [Age, Long, Household] | -3280 |

In bold: best models

## Data dictionary

The data used in the analysis can be found in the data.csv. A description of the variables can be found below

| Variable | Explanation |
| --- | --- |
| participant_id | Unique identification number for each participant |
| cluster_id | Unique identification number for each cluster from which individuals were sampled |
| agecat_participant | Age categories of study participants, as reported in the manuscript |
| day | Week day preceding the survey day, about which information on contacts was asked as per definition (see Methods) |
| agecat_contact | Age categories for contacts, as reported in the manuscript. |
| relationship | Refers to how participant and their contact are linked |
| contact_type | Physical (i.e. skin-to-skin touch) or non-physical |
| contact_duration | Total time spent by the study participant with the named contact during the day preceding the survey day |
| number_casual_contacts | Estimates number of casual contacts (i.e. <5 minutes long) |
| ARS | Presence of acute respiratory symptoms in the last two weeks |
| Carriage | Nasopharyngeal carriage of S.pneumoniae (Yes/No) |
